# Role of CYP2C9, CYP2C19 and EPHX Polymorphism in the Pharmacokinetic of Phenytoin: A Study on Uruguayan Caucasian Subjects

**DOI:** 10.3390/ph10030073

**Published:** 2017-08-18

**Authors:** Natalia Guevara, Cecilia Maldonado, Manuel Uría, Raquel González, Manuel Ibarra, Silvana Alvariza, Antonella Carozzi, Carlos Azambuja, Pietro Fagiolino, Marta Vázquez

**Affiliations:** 1 Pharmaceutical Sciences Department, Faculty of Chemistry, Universidad de la República, 11800 Montevideo, Uruguay; nguevara@fq.edu.uy (N.G.); cmaldonado@fq.edu.uy (C.M.); mibarra@fq.edu.uy (M.I.); salvariza@fq.edu.uy (S.A.); pfagioli@fq.edu.uy (P.F.); 2 Genia-Genetics Molecular Laboratory, Bulevar General Artigas 922, 11300 Montevideo, Uruguay; uria@genia.com.uy (M.U.); carozzi@genia.com.uy (A.C.); azambuja@geniageo.com (C.A.); 3 Toxicology Department, “Dr. Manuel Quintela” Clinical Hospital, Universidad de la República, 11609 Montevideo, Uruguay; rgonzalez@hc.edu.uy

**Keywords:** phenytoin, p-hydroxyphenytoin, CYP2C9, CYP2C19, epoxide hydrolase, polymorphisms, pharmacogenetics

## Abstract

Phenytoin (PHT) oxidative route leads to its main metabolite p-hydroxyphenytoin (p-HPPH), by means of CYP2C9 and CYP2C19. Formation of p-HPPH proceeds via a reactive arene-oxide intermediate. This intermediate can also be converted into PHT dihydrodiol by microsomal epoxide hydrolase (EPHX). The three enzymes are polymorphically expressed and the genetic variants are responsible for changes in the enzyme activity. In order to evaluate the effect that these polymorphisms have on PHT metabolism, PHT and p-HPPH plasma concentrations were measured and the genotype for the three enzymes was assessed in 50 Uruguayan epileptic patients. 30% of the patients were intermediate and 2% were poor metabolizers for CYP2C9, while 20% were intermediate metabolizers for CYP2C19. 44%, 10%, and 46% of subjects had intermediate, increased and decreased activities of EPHX respectively. CYP2C9 was confirmed to be the main responsible enzyme for PHT biotransformation. CYP2C19 seemed to be preponderant in p-HPPH oxidative metabolism. Apart from being responsible for the production of the dihydrodiol metabolite, EPHX also seemed to contribute to pHPPH formation when its activity is low. PHT might be recovered with a decreased activity of EPHX regardless the activity of CYP2C9.

## 1. Introduction

Phenytoin (PHT) is a widely prescribed antiepileptic drug approved for use to treat several types of seizures. PHT biotransformation has been reported to occur through several parallel metabolic pathways, of which the main one is *para*-hydroxylation to form the inactive metabolite p-hydroxyphenytoin (p-HPPH). Its formation goes through an unstable arene-oxide intermediate, and the enzymes involved in this metabolic step are CYP2C9 and CYP2C19, the former accounting for the 90% of *para*-hydroxylation. Most of the p-HPPH formed is then conjugated with glucuronide by uridine-5-diphospho-glucuronosyltransferase 1A isoenzyme (UGT1A) to form hydroxyphenytoin-*O*-glucuronide that is then excreted into urine. p-HPPH can also suffer further oxidation by CYP2C19, 2C9 and 3A4 leading to catechol formation. Despite the fact that p-HPPH accounts for 60–88% of the administered PHT, the arene-oxide intermediate can also be converted into PHT dihydrodiol through microsomal epoxide hydrolase (EPHX), which represents an additional 7–11%. The dihydrodiol can be converted into catechol or conjugated with glucuronide. Less than 5% of the administered dose of PHT is excreted unchanged in urine [1,2,3,4]. 

Other minor metabolites have also been described, such as phenytoin-N-glucuronide, 4,4′-dihydroxyphenytoin (the derivative *p*-hydroxylated on both phenyl rings), a metabolite resulting from the hydantoin ring scission, and *meta*-hydroxyphenytoin. However, this *m*-isomer seems to be an analytical artefact produced during the acid hydrolysis of urine, which is carried out to separate primary metabolites from the glucuronide conjugates, rather than a natural PHT metabolite [2,4,5].

Most of the enzymes involved in PHT metabolism such as CYP2C9, CYP2C19, and EPHX are polymorphically expressed. Regarding CYP enzymes, at least 60 and 35 variant alleles have been identified for CYP2C9 and CYP2C19, respectively [6]. The most common allelic variants for both enzymes are the denominated *2 and *3, which are consequence of single nucleotide polymorphisms (SNP) for CYP2C9 and single point mutation for CYP2C19, both on chromosome 10 [3,7,8,9]. 

CYP2C9*2 is caused by a C > T transition at the base pair 430 (rs 1799853) in exon 3, which leads to the substitution of arginine to cysteine at the amino acidic residue 144 (Arg144Cys). An A > C transversion at the base pair 1075 in exon 7 (rs 1057910) that results in an isoleucine to leucine change at the codon 359 (Ile359Leu), defines the allelic variant CYP2C9*3 [3,8,9,10,11,12]. On the other hand, the polymorphic isoforms CYP2C19*2 and CYP2C19*3 are caused by a G > A transition at the base pairs 681 in exon 5 (rs 4244285) and 683 in exon 4 (rs 4986893), respectively. 681G > A mutation determines an aberrant splicing, resulting in an altered reading frame of the mRNA and a premature protein synthesis termination leading to a non-functional enzyme. 683 G > A mutation determines a premature stop codon, which leads to a truncated protein [2,9,10,12,13]. 

The reduction in CYP2C9*2 catalytic activity observed when compared with the wild-type is a consequence of a reduction in the maximum elimination rate (Vmax). However, for the *3 allelic variant, the catalytic activity is significantly reduced not only because of a decrease in Vmax, but also a decrease in the substrate affinity, which means an increase in Km [7,8,9,14,15].

The combination of *2 and *3 alleles produces six different genotypes for each enzyme, and according to it, the individuals can be classified into normal metabolizers (NM), intermediate metabolizers (IM) or poor metabolizers (PM). Individuals that are homozygous for the wild-type allele (*1/*1) have normal enzyme activity and are classified as NM. However, individuals that carry at least one mutated allele have a decrease in the enzyme activity. Those who are heterozygous, carrying one wild-type allele and one mutant allele (*1/*2 or *1/*3) have a mild-to-moderate decrease in the enzyme activity and are classified as IM, whereas the individuals that are homozygous carrying two mutant alleles (*2/*2, *2/*3 or *3/*3) have a significant decrease in the enzyme activity and are classified as PM [1,16].

The gene that encodes EPHX has been localized to 1p11-qter chromosome and several mutations have been described. However, only two of them were considered to present genetic polymorphisms in Caucasians [9,17,18]. A T > C transition in exon 3 that results in a tyrosine to histidine change in residue 113 (Tyr113His) causes approximately 50% decrease in enzyme activity. On the other hand, an A > G transition in exon 4 that causes the substitution of histidine to arginine at codon 139 (His139Arg), has been associated with an increase by 25% in the enzyme activity [9,17,19]. Three phenotypes have been described taking into account the allele combinations. Subjects that present the wild type allele in homozygosity as well as the ones that are heterozygous for both allelic variants, have an intermediate enzyme activity. However, a decrease in enzyme activity appears with His113 homozygosity or His113 heterozygosity (mutated allele in exon 3) combined with His139 homozygosity (wild-type allele in exon 4), and an increase in enzyme activity occurs with Arg139 homozygosity or Arg139 heterozygosity (mutated allele in exon 4) combined with Tyr113 homozygosity (wild-type allele in exon 3) [20].

PHT is characterized for a non-linear pharmacokinetic, narrow therapeutic range and acute dose-related side effects, which range from gingival overgrowth to cutaneous rashes. This may explain why therapeutic drug monitoring has become a useful tool for clinicians. Nevertheless, polymorphisms in CYP2C9, CYP2C19 and EPHX which are responsible for changes in enzyme activity are rarely taken into consideration and they may be clinically important in treatments with this drug. In fact, in a pharmacokinetic study in healthy volunteers comparing two different dosage regimes of PHT, cutaneous reactions were detected in some subjects [21]. The authors reported that an increase in the production or a decrease in the detoxification of arene-oxide during PHT metabolism seemed to be the cause of the adverse drug reaction (ADR) observed. The study of EPHX polymorphisms on these subjects revealed that all the individuals with cutaneous rash presented mutations in the EPHX. Therefore, as it was stated by the authors, that although several factors could enhance the reactive arene-oxide concentration, one of them is a decrease in EPHX activity. 

Pharmacogenetic studies have shown that genetic defects in drug-metabolizing enzymes encoded by CYP2C9, CYP2C19 may have an important influence on PTH pharmacokinetics. A decrease in CYP2C9 and CYP2C19 catalytic activity might increase PHT concentration and provoke other types of ADRs more related with the central nervous system. Nevertheless, there is little information in the literature about the role of EPHX polymorphism on PHT pharmacokinetics [22,23,24,25]. 

The aim of this study was to assess the effect that CYP2C9, CYP2C19 and EPHX polymorphisms have on PHT metabolism by measuring PHT and p-HPPH plasma concentrations in Uruguayan epileptic patients.

## 2. Results

CYP2C9 and CYP2C19 genotypes and phenotypes frequencies are shown in Table 1 and Table 2, respectively. Twenty-six patients (52.0%) had no mutations in either CYP2C9 or CYP2C19 (*1/*1 for both enzymes), and only two (4.0%) patients had genetic mutations in both enzymes. Eleven (22.0%) and four (8.0%) patients were heterozygous for Arg144Cys (CYP2C9*1/*2) and Ile359Leu (CYP2C9*1/*3), respectively. Therefore, they were classified as IM for CYP2C9. One (2.0%) patient was homozygous for *2 variant of CYP2C9 and classified as PM. The remaining thirty-four patients (68.0%) were classified as NM for this enzyme as they had the *1/*1 genotype. Thus, the allele frequencies of CYP2C9*2 and CYP2C9*3 are 0.13 and 0.04, respectively. On the other hand, forty patients (80.0%) were classified as NM for CYP2C19 since they were homozygous for the wild-type allele (*1/*1). The remaining ten (20.0%) patients were classified as IM owing to the fact that they were heterozygous for *2 variant (CYP2C19*1/*2). No patient was classified as PM for this enzyme since no one was homozygous for *2 allele. No patient was found to exhibit CYP2C19*3 mutation either. Therefore, the allele frequency for CYP2C19*2 is 0.10.

EPHX genotypes and phenotypes frequencies are shown in Table 3, Table 4 and Table 5. Regarding polymorphism in exon 3 (Tyr113His), twenty one (42.0%) patients were homozygous for Tyr113 (wild-type allele), other twenty-two (44.0%) were heterozygous and the remaining seven (14.0%) were homozygous for His113. Considering exon 4 polymorphism (His139Arg), thirty-seven (74.0%) patients were homozygous for His139 (wild-type allele), eleven (22.0%) were heterozygous and the remaining two (4.0%) were homozygous for Arg139. Taking into account these results, twenty-two (44.0%) patients were considered to have intermediate, five (10.0%) patients increased, and twenty-three (46.0%) decreased EPHX enzyme activity. 

Doses were normalized by patients’ weight, and the overall mean dose was 4.388 mg/kg (range 1.852–8.889). PHT and p-HPPH concentrations were obtained in all 50 patients at least in three instances and average concentrations were calculated for each patient. PHT and p-HPPH concentrations were corrected by the normalized dose (from now on [PHT] and [p-HPPH], respectively) in order to test the genotype influence on drug concentration at the same daily dose. Because of this, normalized-by-dose concentration units are kg/L (mg·L^−1^/mg·kg^−1^). Concentration means were 1.789 kg/L (range 0.5279–4.055) and 0.0240 kg/L (range 0.0076–0.0708), for PHT and p-HPPH respectively. 

Means of PHT normalized dose, [PHT] and of [p-HPPH] in each paired CYP2C9/CYP2C19 phenotype group are shown in Table 6. Differences in [PHT] and [p-HPPH] among NM/NM, NM/IM and IM/NM groups were not significant (ANOVA, *p* > 0.2). Comparisons of both [PHT] and [p-HPPH] single values coming from IM/IM group (average of two data) or PM/NM group (single data) with each of the previous groups were evaluated by a simple Student’s *t*-test, assessing whether those values could belong to each respective population. With regards to IM/IM, [PHT] was significantly different from all the three populations (*p* < 0.001), whereas [p-HPPH] was significantly different from the NM/NM population (*p* < 0.01). Concerning the PM/NM single data, [PHT] was significantly different from each of the three populations (*p* < 0.001), whereas the [p-HPPH] was significantly different from NM/NM and from IM/NM populations (*p* < 0.01).

Means of PHT normalized dose, [PHT] and [p-HPPH] for CYP2C9, CYP2C19 and EPHX phenotypes are shown in Table 7. The differences in [PHT] and [p-HPPH] were studied keeping one variable fixed. This means that the phenotype of at least one of the enzymes was the same among the studied groups. 

To study the effect of EPHX on PHT-arene-oxide metabolism, only [p-HPPH] should be analysed because differences in EPHX activity might only change the concentration of products coming from the precursor molecule. For both CYPs normal metabolizers no significant difference was found among the different groups in accordance with the activity of EPHX (ANOVA, *p* = 0.5). Even though [PHT] means were not significantly different, their values spread along a wide range in the following order: decreased or intermediate > increased regarding EPHX phenotype. 

To study the effect of both CYPs on PHT and p-HPPH metabolism, the groups with differences in CYPs phenotypes but with the same EPHX phenotype were compared. The NM/NM was chosen as the reference group whatever the activity of EPXH was. Hence, and regarding the intermediate EPHX phenotype, only the PM/NM group had significant difference for both [PHT] and [p-HPPH] (*p* < 0.01). Considering the increased EPHX activity, the IM/IM group showed significant difference only for PHT (*p* < 0.01). Finally, taking into consideration the decreased EPHX activity, only the IM/IM group differed significantly with the reference in [PHT] (*p* < 0.001).

## 3. Discussion

CYP2C9 genotype frequencies found in this study resemble the frequencies reported among Caucasian populations by Lee et al. [7]. Nevertheless, CYP2C19*2 frequency appeared to be higher than those reported for Caucasian but lower than dose reported for Asian population by Alonso-Navarro et al., [26]. It should be noted that CYP2C19*3 was not found in this Uruguayan population. An allelic variant of CYP2C19 that has been associated with an increase in the enzyme activity is the denominated *17. Although few studies have been conducted including this variant, the allelic frequencies found were 18% in Swedes, 19.6% in Greeks, 18% in Ethiopians and 4% in Chinese. However, controversial results have been reported with regards to the magnitude of the effect CYP2C19*17 may have. Taking this into consideration and in addition to the fact that, as it was mentioned in the introduction section, CYP2C9 accounts for 90% of *para*-hydroxylation, CYP2C19*17 variant was not considered in this study [27,28,29,30].

With regards to EPHX, the genotype frequency found in this study for Tyr113 heterozygous was similar, while for His113 homozygous was higher than those reported among Caucasian population. His139 heterozygous and Arg139 homozygous frequencies were lower and similar, respectively, than those reported among Caucasians [9,17]. 

Comparisons in [PHT] were carried out between the different paired CYP2C9/CYP2C19 phenotypes. NM/IM and IM/NM groups, in which there is a mild-to-moderate decrease in CYP2C19 and CYP2C9 enzyme activity, respectively, were compared between them and with the one with the highest enzyme activity (wild-type NM/NM). Although no significant difference was obtained in any of the comparisons, it should be noted that [PHT] in the NM/IM group remained unchanged despite the decrease in CYP2C19 activity, while the IM/NM group had higher [PHT] not only when compared with the NM/NM group (18% higher), but also with the NM/IM group (17% higher). According to these results, it can be said that PHT metabolism may not be affected when only CYP2C19 activity is decreased owing to the fact that CYP2C9, which has been reported to have a predominant role in PHT metabolism [4], is able to compensate the decrease in CYP2C19 activity. On the other hand, when CYP2C9 activity is decreased, CYP2C19 is not able to compensate it and an increased [PHT] is seen, because of a reduced PHT clearance. These observations confirm a secondary role of CYP2C19 in PHT metabolic pathway. 

However, when the IM/IM group was analysed, [PHT] was found to be considerably higher (81% regarding NM/NM), showing significant difference (*p* < 0.001) when compared with the wild-type phenotype and with the groups in which only one of the enzymes had a decreased activity. These results show a drastic reduction in the PHT clearance when both CYPs diminish their activities, reinforcing the idea that CYP2C19 has a minor role in PHT metabolism.

[p-HPPH] was also compared among the CYP2C9/CYP2C19 phenotypes, and an increase was seen when the NM/IM group was compared with the NM/NM group. This increase in [p-HPPH], even not significant, can be attributed to a reduction in the clearance of the p-HPPH → catechol route, which is catalysed by CYP2C19 among other CYPs. In addition, the fact that [PHT] remained unchanged, evidenced that CYP2C19 may be more involved in p-HPPH than in PHT biotransformation. Moreover, CYP2C9 was not able to compensate the decrease in CYP2C19 activity, giving some evidence about the secondary role CYP2C9 could have in the p-HPPH biotransformation. A higher number of subjects would be necessary to confirm the tendency here shown.

It should be kept in mind that the metabolite concentration depends not only on its clearance, but also on its bioavailability. The single case of a woman having PM/NM phenotype, who showed a considerable decrease in CYP2C9 enzyme activity, reinforces our suspicion. Despite being a normal metabolizer for CYP2C19, she appeared to have the highest [PHT] (significant increase of 93% regarding the NM/NM group). As CYP2C19 was not able to metabolise PHT like CYP2C9, a decrease in PHT clearance must produce the highest PHT level, even higher than in the IM/IM group. However, the significant decrease in [p-HPPH] (61% when compared with NM/NM group) could only be compatible with a lower bioavailability of p-HPPH since its clearance should remain unchanged because of the constancy in CYP2C19 activity (NM and NM). 

Once again, IM/IM group showed a 68% decrease in [p-HPPH] when it was compared with the NM/NM wild-type phenotype (*p* = 0.01). Here, a decreased bioavailability of p-HPPH should be the cause since its clearance is supposed to be even lower than in the wild-type group. Interestingly, PM/NM and IM/IM had intermediate and increased activity of EPHX, respectively.

In order to assess the role that EPHX plays in p-HPPH bioavailability, metabolite plasma concentrations were analysed taking into consideration the phenotype of the three enzymes all together: CYP2C9, CYP2C19 and EPHX. Once the arene-oxide is formed, it has two possible routes of elimination: p-HPPH or dihydrodiol formation. If the enzyme leading to the dihydrodiol formation (EPHX) has an increased activity, a decrease in the p-HPPH formation route and thus in [p-HPPH] is expected. Conversely, if EPHX activity is decreased, dihydrodiol formation is reduced, leading to an increase in [p-HPPH].

No significant differences were found in [p-HPPH] among the three groups in which the phenotype for both CYPs was NM but EPHX activity varied from increased to intermediate, and to decreased. However, a 66 % decrease in mean [p-HPPH] was observed in the NM/NM/Increased group in relation with NM/NM/Intermediate wild-type group. Maybe, the number of subjects was not enough to reach significance despite the great difference between means. On the other hand, a 6% increased level of p-HPPH was seen in the NM/NM/Decreased group when compared with the wild-type phenotype. These results do not confirm but support the EPHX role in the p-HPPH exposure. 

**Figure 1:**
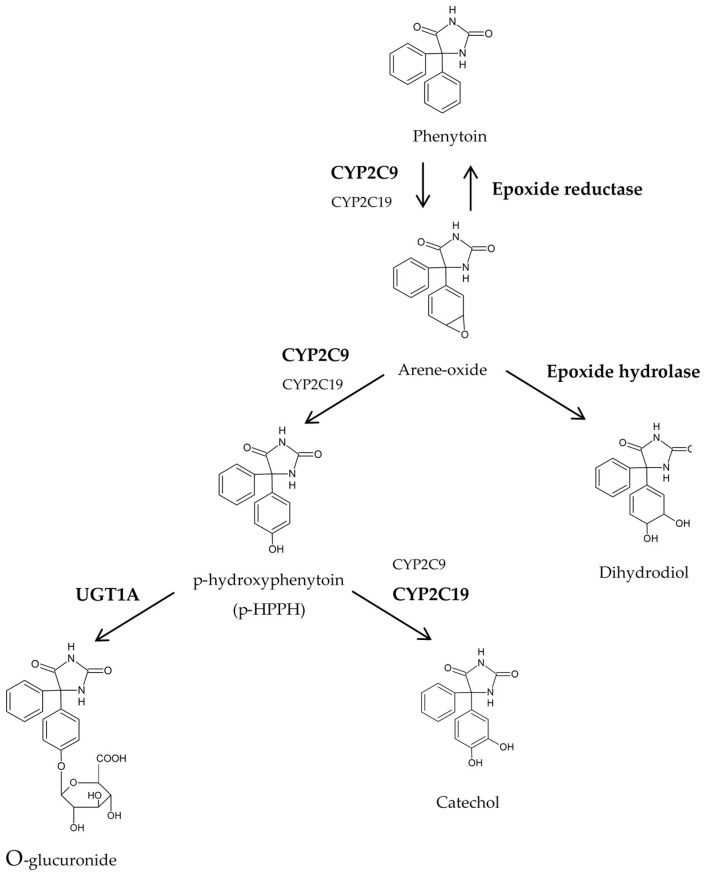
Part of the phenytoin biotransformation pathway.

Some interesting findings regarding [PHT] deserve to be reported as well. Even though [PHT] means were not significantly different, their values spread along a wide range, in the following order: decreased or intermediate > increased regarding EPHX phenotype. This could be revealing some kind of reversible formation of the arene-oxide, by which the parent drug PHT might be recovered when the intermediate metabolite is impeded of being transformed. Although this pathway has never been reported for PHT, the reduction of arene-oxide back to the parent compound was first studied by Booth et al., [31]. Analyzing the metabolism of benz[a]anthracene in rats, the authors proved that the reduction of benz[a]anthracene 5,6-oxide to the parent hydrocarbon took place, being the microsomal epoxide reductase the enzyme that mediated the reaction. Moreover, the authors reported that the amount of parent hydrocarbon formed by reduction of the arene-oxide was increased under anaerobic conditions or when an EPHX inhibitor such as cyclohexene oxide was added. Therefore, epoxide reductase participation in the metabolism of PHT-arene-oxide should not be neglected. Part of PHT biotransformation pathway described in the Introduction section could be now supplemented with our findings as shown in Figure 1.

In conclusion, CYP2C9 appears to have a predominant role in PHT biotransformation and a minor role in p-HPPH biotransformation, since it is not able to metabolize p-HPPH to the same extent that CYP2C19 does. On the other hand, CYP2C19 seems to have a predominant role in p-HPPH phase-I biotransformation and a minor role in PHT biotransformation, since it is not able to metabolize PHT as CYP2C9. It should be taken into account that p-HPPH also follows a phase-II metabolism, but this route of elimination was not genotyped. Finally, EPHX seems to determine the bioavailability of p-HPPH. The limitation of this study is the low number of patients included to have sufficient statistical power. However, this number is representative of the low number of patients under PHT monotherapy or with other non-inducer antiepileptic drugs.

## 4. Materials and Methods 

### 4.1. Subjects and Design 

A total of fifty Caucasian patients (25 female, 25 male) with epilepsy between 18 and 76 years old, mean body weight (±SD) of 74.48 kg (±16.15) and under chronic treatment with oral PHT were enrolled in this study. Patients with hepatic or renal impairment were excluded. The study protocol was designed according to the clinical research guidelines and was approved by the Institutional Ethics Review Committee of the Faculty of Chemistry (Universidad de la República, Montevideo, Universidad de la República, Montevideo, Uruguay). Written informed consent was obtained from all subjects before their entry in the study. 

### 4.2. Genotyping Procedure of EPHX, CYP2C9 and CYP2C19

Blood sample (2 mL) was collected by venipuncture and placed in sterile, siliconised, ethylenediaminetetraacetic acid (EDTA) tubes. Immediately after collection, blood was stored in a refrigerator (4–8 °C) until analysis. Genomic DNA was isolated from whole blood using the Wizard^®^ genomic DNA purification kit, according to the manufacturer´s instructions (Promega, Madison, WI, USA). Then, it was quantified by spectrophotometry (260/280 nm) on a NanoQuant-Tecan instrument (Tecan, Männedorf, Switzerland) and, according to this quantification, a dilution was performed in order to put 3 to 20 ng in the amplification mix for the EPHX genotyping protocol, and between 50 and 100 ng for the CYP genotyping protocol. To determine the genotype of EPHX a real time polymerase chain reaction (RT-PCR) protocol was used and a TaqMan Drug Metabolism Genotyping Assay (Applied Biosystems, Foster City, CA, USA) for rs 1051740 and rs 2234922 performed in StepOne (Applied Biosystems). To determine CYP2C9 and 2C19 genotype, a conventional PCR was performed for each SNP (rs 1799853 and rs 1057910 for CYP2C9; rs 4244285 and rs 4986893 for CYP2C19). The PCR product was verified by gel electrophoresis in polyacrylamide 6% and 5 μL of the PCR product were purified and sequenced with specially designed primers and BigDye^®^ (Thermo Fisher Scientific Inc., Waltham, MA, USA) Terminator v3.1 Cycle Sequencing Kit (Applied Biosystems). Then, the product was purified with BigDye^®^ X-Terminator Purification Kit (Applied Biosystems) and a capillary electrophoresis in an ABI3500 sequencer (Applied Biosystems) was performed.

### 4.3. Measurement of PHT and p-HPPH Plasma Concentrations

Morning pre-dose blood samples (5 mL) were withdrawn by venipuncture and immediately placed into heparinized tubes. Plasma was separated by centrifugation and stored at −20 °C until analysis. PHT and p-HPPH plasma concentrations were determined by a high performance liquid chromatography (HPLC) method based on a procedure previously published by Savio et al., with minor modifications [32]. Sample preparation involved extraction of PHT and p-HPPH with 3 mL of ethyl acetate from 0.5 mL of plasma, evaporation of the organic phase under a nitrogen stream at 40 °C and reconstitution of the dry residue with 100 µL of mobile phase. Fifty microliters of a nitrazepam solution (16 mg/L in methanol) were used as internal standard. Macherrey-Nagel Nucleodur C18 (5 µm, 150 mm × 4.6 mm) column (Macherey-Nagel, Düren, Germany) was used as a reversed stationary phase. The mobile phase consisted of a mixture of water/methanol/acetonitrile (43:47:10) pumped with a flow rate of 1.0 mL/min. The column compartment was kept at 40 °C, the wavelength detection was 220 nm and the injection volume was 20 μL. Under these conditions, the retention times of analytes were 2.5, 4.5 and 5.8 min for p-HPPH, PHT and nitrazepam respectively. The HPLC method was linear between 0.5286 mg/L (the lower limit of quantification, LLOQ) and 24.39 mg/L for PHT and between 0.0585 mg/L (LLOQ) and 2.701 mg/L for p-HPPH. Within-day and between-day precisions (coefficient of variation: CV) for low, intermediate, and high concentrations of the calibration curve were below 15% and the accuracy of the method was between 85–115% for both analytes. 

### 4.4. Statistical Analysis

All statistical analyses were carried out using SPSS (version 17.0 for Windows). The significances of differences were calculated by performing analysis of variance (ANOVA) or a Student’s *t*-test as the data was normally distributed. In the case of a single value or an average of two values, a Student’s *t*-test was applied in order to know if these values belong to a given population. All *p*-values were two sided, using α = 0.05 as the reference standard for determining the significance.

## Figures and Tables

**Table 1:** Genotype and phenotype frequencies for CYP2C9.

CYP2C9 Genotype	N° of Subjects	Percentage	CYP2C9 Phenotype	N° of Subjects	Percentage
*1/*1	34	68.0	NM	34	68.0
*1/*2	11	22.0	IM	15	30.0
*1/*3	4	8.0			
*2/*2	1	2.0	PM	1	2.0

**Table 2:** Genotype and phenotype frequencies for CYP2C19.

CYP2C19 Genotype	CYP2C19 Phenotype	N° of Subjects	Percentage
*1/*1	NM	40	80.0
*1/*2	IM	10	20.0

**Table 3:** Genotype frequencies for EPHX exon 3 polymorphism.

Genotype	N° of Subjects	Percentage
A/A wild-type (Tyr113Tyr)	21	42.0
A/G heterozygous (Tyr113His)	22	44.0
G/G mutated homozygous (His113His)	7	14.0

**Table 4:** Genotype frequencies for EPHX exon 4 polymorphism.

Genotype	N° of Subjects	Percentage
T/T wild-type (His139His)	37	74.0
T/C heterozygous (His139Arg)	11	22.0
C/C mutated homozygous (Arg139Arg)	2	4.0

**Table 5:** Phenotype frequencies for EPHX.

Phenotype	N° of Subjects	Percentage
Intermediate	22	44.0
Increased	5	10.0
Decreased	23	46.0

**Table 6:** Means (95% confidence interval) for PHT normalized dose, and for PHT and p-HPPH dose-corrected concentrations, according to the paired CYP2C9/CYP2C19 phenotype.

CYP2C9 Genotype	CYP2C19 Genotype	N° of Subjects	CYP2C9/CYP2C19 Phenotype	PHT Normalized Dose (mg/kg)	[PHT] (kg/L)	[p-HPPH] (kg/L)
*1/*1	*1/*1	26	NM/NM	4.545 (3.771–4.938)	1.628 (1.207–2.050)	0.0250 (0.0190–0.0309)
*1/*1	*1/*2	8	NM/IM	4.5817 (3.3811–5.7823)	1.644 (1.083–2.204)	0.0294 (0.0154–0.0434)
*1/*2	*1/*1	9	IM/NM	4.0893 (3.5590–4.6196)	1.917 (1.402–2.431)	0.0206 (0.0169–0.0243)
*1/*3		4				
*1/*2	*1/*2	1	IM/IM	4.0813 (3.0489–5.1136)	2.943 (2.862–3.023)	0.0170 (−0.0194–0.0533)
*1/*3		1				
*2/*2	*1/*1	1	PM/NM	3.2258	3.140	0.0153

Note: IM/IM group showed significant difference in [PHT] with respect to NM/NM, NM/IM and IM/NM groups (*p* < 0.001) and in [p-HPPH] with respect to NM/NM group (*p* < 0.01). PM/NM group showed significant difference in [PHT] with respect to NM/NM, NM/IM and IM/NM groups (*p* < 0.001) and in [p-HPPH] with respect to NM/NM and IM/NM groups (*p* < 0.01).

**Table 7:** Means (95% confidence interval) for PHT normalized dose, and for PHT and p-HPPH dose-corrected concentrations, according to CYP2C9/CYP2C19/EPHX phenotype.

CYP2C9/CYP2C19/EPHX Phenotype	N° of Subjects	Dose (mg/kg)	[PHT] (kg/L)	[p-HPPH] (kg/L)
NM/NM/Intermediate	10	4.279 (3.446–5.112)	1.886 (1.033–2.740)	0.0256 (0.0193–0.0319)
NM/NM/Increased	4	5.391 (1.535–9.247)	1.020 (0.1884–1.851)	0.0168 (0.0073–0.0263)
NM/NM/Decreased	12	4.485 (3.569–5.401)	1.616 (0.9670–2.266)	0.0271 (0.0146–0.0397)
NM/IM/Intermediate	4	4.346 (1.655–7.037)	1.420 (0.7667–2.072)	0.0325 (−0.0065–0.0715)
NM/IM/Decreased	4	4.817 (2.680–6.955)	1.868 (0.4940–3.241)	0.0263 (0.0187–0.0339)
IM/NM/Intermediate	7	4.213 (3.161–5.264)	1.673 (0.8725–2.474)	0.0223 (0.0158–0.0288)
IM/NM/Decreased	6	3.945 (3.421–4.470)	2.201 (1.349–3.052)	0.0187 (0.0137–0.0237)
IM/IM/Increased	1	4.000	2.936	0.0141
IM/IM/Decreased	1	4.163	2.949	0.0198
PM/NM/Intermediate	1	3.226	3.140	0.0153

Notes: PM/NM/Intermediate showed significant difference in [PHT] and [p-HPPH] with respect to NM/NM/Intermediate (*p* < 0.01). IM/IM/Increased showed significant difference in [PHT] with respect to NM/NM/Increased (*p* < 0.01). IM/IM/Decreased showed significant difference in [PHT] with respect to NM/NM/Decreased (*p* < 0.001).
